# Acute Post-streptococcal Glomerulonephritis in Children: A Moroccan Experience

**DOI:** 10.7759/cureus.94683

**Published:** 2025-10-15

**Authors:** Samira Tizki, Houda Nassih, Rabiy Elqadiry, Aicha Abourrahouat, Laila Lahlou, Imane Aitsab

**Affiliations:** 1 Pediatrics, University Hospital Center Souss Massa, Faculty of Medicine and Pharmacy, Ibn Zohr University, Agadir, MAR; 2 Pediatrics, Mother and Child Hospital, Mohammed VI University Hospital, Faculty of Medicine and Pharmacy, Cadi Ayyad University, Marrakech, MAR; 3 Laboratory of Health Sciences, Faculty of Medicine and Pharmacy, Ibn Zohr University, Agadir, MAR

**Keywords:** acute kidney injury, acute post-streptococcal glomerulonephritis, nephritic syndrome, pediatrics, rapidly progressive glomerulonephritis, renal outcome

## Abstract

Background: Acute post‐streptococcal glomerulonephritis (APSGN) is the most prevalent form of glomerulonephritis in children. It is caused by an immune‐mediated glomerular injury, most commonly secondary to Group A β-hemolytic streptococcus. This study aims to describe the characteristics and outcomes of hospitalized children with APSGN.

Methods: This was a retrospective descriptive study of 83 pediatric patients (<15 years) admitted to the pediatric nephrology department in the Mohamed VI Hospital in Marrakech with APSGN between January 2019 and December 2023, with a follow-up of one year. Children with acute glomerulonephritis not related to APSGN were excluded.

Results: The mean age was 8.34 ± 3.58, and 69.9% (n = 58) were male. Among the 83 APSGN patients, 17 (20.4%) progressed to rapidly progressive glomerulonephritis (RPGN). The most often given presentations were hematuria (90.3%, n=75), edema (77.1%, n=64), hypertension (66.3%, n=55), and oliguria (12%, n=10). 15.6% (n=13) of the patients had an acute kidney injury (AKI). Nephrotic syndrome was found in 33.7% (n=28) of children, reduced C3 level in 84.3% (n=70), and elevated antistreptolysin O titer (ASOT) in 69.9% (n=58). Kidney biopsy was performed in 50.6% of children (n=42). 38.6% (n=32) of our patients required two anti‑hypertensives such as furosemide and nicardipine. Kidney replacement therapy was required for 6% (n=5) of patients, antibiotics for 36.1% (n=30), methylprednisolone pulses for 56.6% (n=47), and cyclophosphamide pulses for 20.4% (n=16). The outcome was favorable for all children, except for one girl with RPGN who developed a chronic kidney disease.

Conclusions: APSGN is still one of the most frequent causes of glomerulonephritis in Morocco. The main presenting features were hematuria and hypertension. Although the outcome of APSGN is good, sequential follow-up is necessary to detect long-term complications and prevent morbidity and mortality.

## Introduction

Glomerular diseases are a primary cause of kidney failure that affects 10-15% of children [1]. Acute post‐streptococcal glomerulonephritis (APSGN) is the most prevalent form of glomerulonephritis in children. An immune‐mediated glomerular injury causes it, most commonly secondary to Group A β-hemolytic *Streptococcus* [2]. APSGN remains one of the important causes of pediatric hospital admission for acute kidney disease (AKD) [1]. Its incidence has declined over the last few decades in many developed countries (0.3 new cases per 100,000 individuals per year); however, in low-income countries, the incidence is estimated to be much higher (9.5 to 28.5 new cases per 100,000 individuals per year) [3]. According to the World Health Organisation, 470,000 new cases of APSGN worldwide are estimated each year, 97% of them in less affluent areas [4]. The healthcare system is severely impacted by the high frequency of APSGN in underdeveloped nations like Morocco, where limited access to primary care, poor infection control measures, and late diagnosis make the disease burden higher. In addition to direct healthcare costs, the economic burden includes lost school days, less productive parents, and long-term monitoring for possible chronic kidney disease in affected children.

Although APSGN has a low rate of mortality (1%) [5], it can sometimes lead to serious complications such as congestive heart failure, pulmonary edema, encephalopathy, and hypertensive emergency [6]. Still, the most severe renal disorder is the rapidly progressive glomerulonephritis (RPGN), which can lead in a few weeks or months to end-stage renal failure [6].

This study aims to present five years of admissions data to describe the characteristics and outcomes of hospitalized children with APSGN at the University Hospital Mohammed VI, Marrakech, to give useful data to improve clinical practice and minimize the burden that APSGN adds to the Moroccan healthcare system.

## Materials and methods

Study design and data collection

We conducted a retrospective descriptive study of pediatric patients admitted to the pediatric nephrology department at Mohamed VI Hospital in Marrakech with a diagnosis of APSGN between January 2019 and December 2023.

Children under 15 years of age were included with a one-year follow-up if they presented with signs of APSGN, defined as: 1) Acute onset of glomerulonephritis (hematuria and/or proteinuria); 2) Hypocomplementemia (serum C3 levels below the normal reference range for age); 3) Evidence of recent group A streptococcal infection, confirmed by at least one of the following: antistreptolysin O (ASO) titer > 200 IU/mL, positive anti-DNase B test, and a throat culture that tested positive for Streptococcus pyogenes.

The exclusion criteria include neonates, patients with acute glomerulonephritis due to causes other than APSGN (such as lupus nephritis and IgA nephropathy), and those with incomplete medical records.

Clinical data extraction

Data were extracted from hospital medical records using a standardized data abstraction form designed for this study. Variables collected included demographics (age, sex, race/ethnicity), clinical features at admission (blood pressure, presence of edema, gross hematuria), laboratory parameters (urea, serum creatinine, total protein, serum albumin, complement (C3, C4), ASO titers, and urine protein quantification), treatment details, including antihypertensive medications, diuretics, and the need for renal replacement therapy, and outcomes (length of hospital stay, complications, and renal function at follow-up).

Height and serum creatinine were used to calculate estimated glomerular filtration rate (eGFR) via the Schwartz formula.

Ethical considerations

This was a retrospective study using anonymized patient records; the requirement for individual informed consent was waived.

Statistical analysis

Statistical analysis was performed using Jamovi version 2.3.28. Normally distributed data were presented as mean ± standard deviation (SD), and skewed distributed data were presented as the median and interquartile range (IQR). Counting data were presented as a rate or percentage. The chi-squared test, as applicable, was used to compare categorical variables. The Shapiro-Wilk test was used to determine if continuous variables were normal, and the independent samples t-test or the Mann-Whitney U test, depending on the data distribution, was used to compare groups. Statistical significance was defined as a P-value of less than 0.05. Results were presented as tables.

Definitions

Hypertension was defined according to the 2017 American Academy of Pediatrics (AAP) Guideline for Childhood Hypertension [7]. For children aged one to 13 years, Elevated ≥90th percentile, Stage 1 ≥95th to <95th percentile + 12 mmHg, Stage 2 ≥95th percentile + 12 mmHg, and Acute Severe ≥95th percentile + 30 mmHg. For children aged ≥13 years, Elevated = 120/<80 to 129/<80 mmHg, Stage 1 = 130/80 to 139/89 mmHg, and Stage 2 ≥140/90 mmHg.

Acute kidney injury (AKI) was defined according to the 2012 Kidney Disease: Improving Global Outcomes (KDIGO) guideline [8]. Changes from baseline serum creatinine (SCr) or urine output (UO) determined the severity of AKI. Stage 1 = SCr 1.5-1.9 times baseline or UO <0.5 mL/kg/h for six to 12 hours, Stage 2 = SCr 2.0-2.9 times baseline or UO <0.5 mL/kg/h for ≥12 hours, and Stage 3 = SCr 3.0 times baseline or UO <0.3 ml/kg/h for ≥24 hours or anuria for ≥12 hours. The normal SCr range for age and sex was utilized when the baseline SCr was unknown.

Oliguria was defined as diuresis < 0.5 mL/kg/hour. Nephrotic syndrome was defined by proteinuria of 50 mg/kg/day (or a proteinuria/creatinuria ratio proteinuria/creatinuria ratio greater than 0.2 g/mmol or >2 g/g), combined with albuminemia of less than 30 g/L. eGFR was calculated according to the Schwartz formula: eGFR (ml/min per 1.73 m²) = 0.413 x (height (cm)/serum creatinine(mg/dL)) [9].

Rapidly progressive glomerulonephritis glomerular disease was defined as proteinuria, hematuria, and red cell casts accompanied by rapid loss of kidney function with rising creatinine over days to weeks [10].

## Results

**Figure 1:**
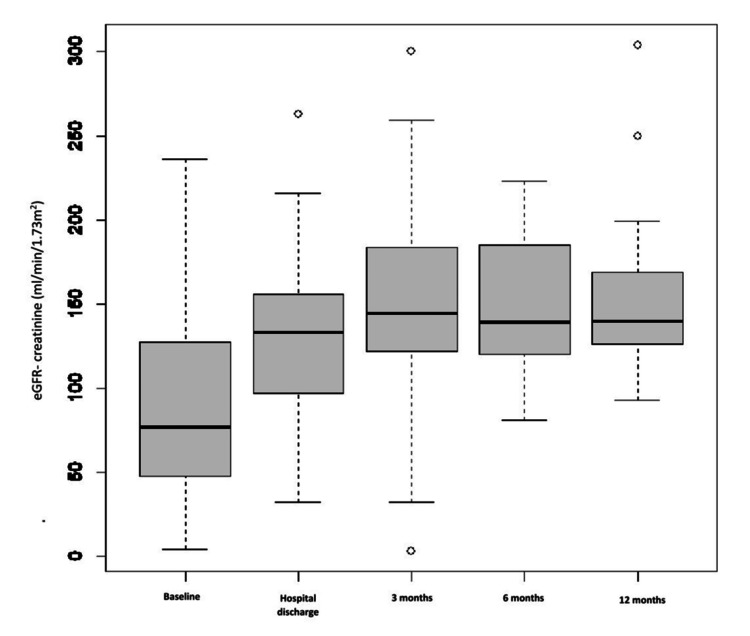
Estimated glomerular filtration rate (eGFR)-creatinine values in the cases measured during the follow-up

**Figure 2:**
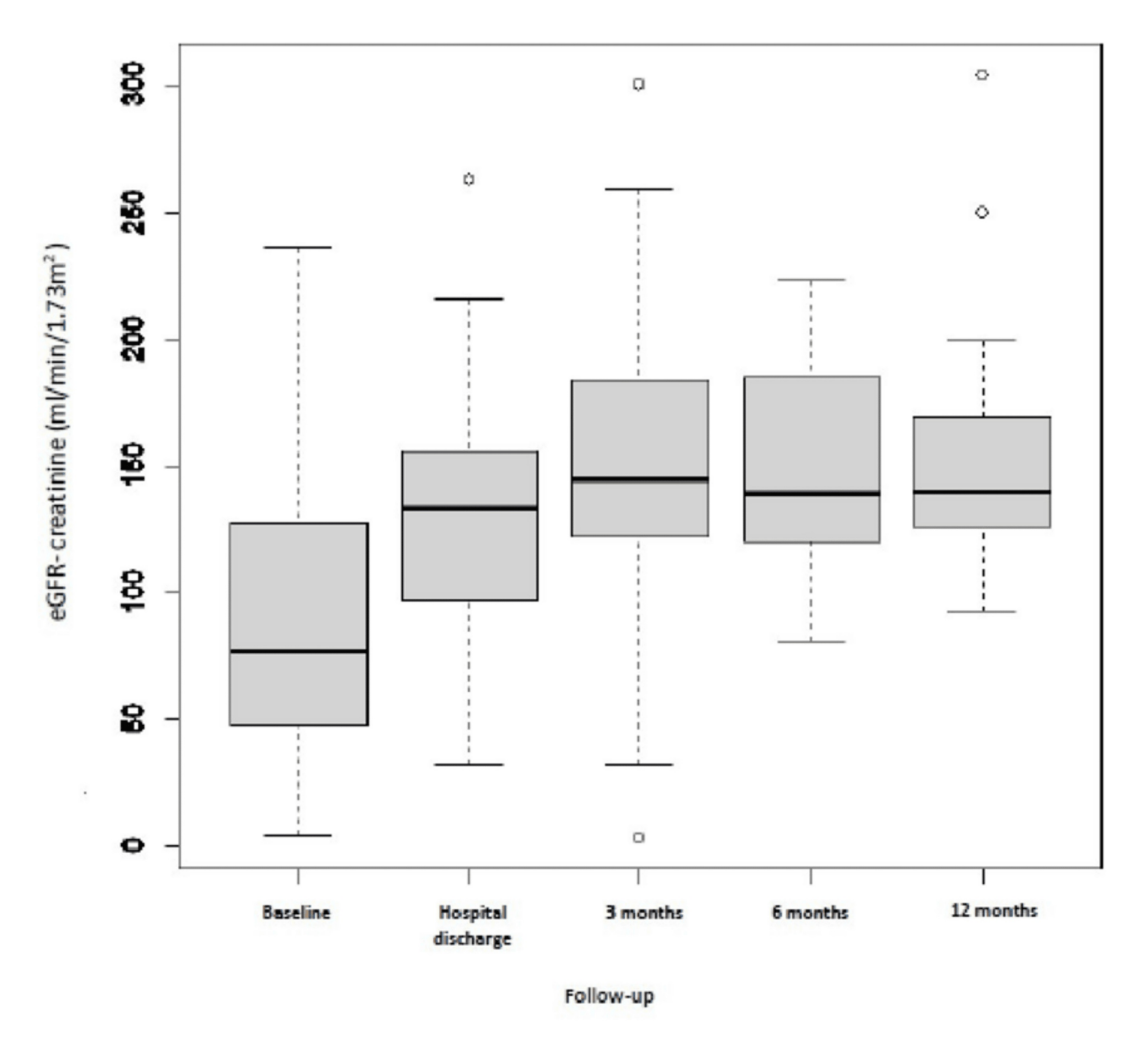
Proteinuria values in the cases measured during the follow-up

Eighty-three children were included in our study; the mean age was 8.34 ± 3.58, and 69.9% were male. 54.2% of children came from a low socioeconomic level. Upper respiratory tract infections (URTIs) accounted for 66.3% as the main source of infection. The interval between the streptococcal infection and the initial APSGN symptoms was 10.8 ± 8.7 days. The seasonal distribution showed a higher incidence of APSGN in winter (41%) and autumn (33.7%). Table 1 summarizes the clinical characteristics. The most often given presentations were hematuria (90.3%), edema (77.1%), hypertension (66.3%), and oliguria (12%). Four of the children had severe symptoms, such as severe hypertension with seizures. At the time of APSGN diagnosis, the mean serum creatinine level was 0.79 mg/dL. 15.6% of the patients had an AKI, with stage 1 AKI severity being 3.6%, stage 2 being 3.6%, and stage 3 being 8.4%. The mean calculated glomerular filtration rate was 86.7 ml/min/1.73 m². Nephrotic syndrome was found in 33.7% of children, the C3 level was reduced in 84.3%, and ASOT was elevated in 69.9%. A kidney biopsy was performed in 50.6% of children for nephrotic syndrome (31.3%) and AKI (16.9%). All of them revealed exudative proliferative glomerulonephritis and C3 on immunofluorescent staining and dome-shaped subepithelial deposits (humps). It also found glomerular crescent in 24.4% (one case with 100% of glomerular crescent and one case of fibrosis). 38.6% of our patients required two antihypertensives, such as furosemide and nicardipine. Kidney replacement therapy was required for 6% of patients, antibiotics for 36.1%, methylprednisolone pulses for 56.6%, and cyclophosphamide pulses for 20.4%. During the acute presentation, no death was noticed, and the median length of hospital stay was eight days. Regarding follow-up (at three months, six months, and 12 months) (Figures 1, 2), the outcome was favorable for all children, except for one girl with RPGN who developed chronic kidney disease (CKD).

**Table 1:** Baseline characteristics of children with acute post‐streptococcal glomerulonephritis (APSGN) at presentation * : number (percentage %), AKI: Acute kidney injury, eGFR: estimated Glomerular Filtration Rate, ASOT: Antistreptococcal antibody titers

Parameter	Value (N = 83)
Age, mean ± SD	8.34 ± 3.58
Sex, n (%): Male	58 (69.9)*
Female	25 (30.1) *
Socio-economic level, n (%): Low	45 (54.2) *
High	38 (45.8)*
Clinical Manifestations (n (%): Oedema	64 (77.1) *
Haematuria	75 (90.3) *
Hypertension	55 (66.3) *
Oliguria	10 (12) *
Source of infection (n (%): Pharyngitis	55 (66.3) *
Skin lesion	15 (18.1)*
Serum creatinine, mg/dL (median [IQR])	0.79 [0.53–1.43]
AKI, n (%)	13 (15.6)*
Stage 1	3 (3.6)*
Stage 2	3 (3.6)*
Stage 3	7 (8.4)*
eGFR, mL/min/1.73 m², mean ± SD	86.7 ± 54.4
Nephrotic proteinuria/syndrome, n (%)	28 (33.7) *
Reduced C3 level, n (%)	70 (84.3) *
Elevated ASOT, n (%)	58 (69.9)*
Kidney biopsy, n (%): Performed	42 (50.6)*
Glomerular crescent	10 (24.4) *
Treatment n (%): Diuretic	75 (90.4) *
Anti-hypertensive	32 (38.6)*
Kidney replacement therapy	5 (6) *
Anti-microbial therapy	30 (36.1)*
Methylprednisolone pulse therapy	47 (56.6) *
Cyclophosphamide pulse therapy	16 (20.4)*
Hospitalization time, days, mean ± SD	8.95 ± 5.38

**Figure 3:**
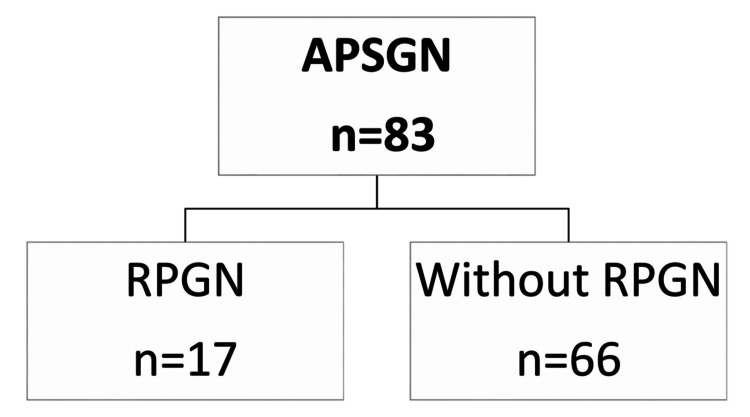
Flow diagram of patients with acute post‐streptococcal glomerulonephritis (APSGN) RPGN: rapidly progressive glomerulonephritis

Among the 83 of our APSGN patients, 17 (20.4%) progressed to RPGN (Figure 3). Seven of them had RPGN at the first presentation. In this group of RPGN, 41.2% were female, in contrast with 27.3% in the group without RPGN (Table 2). At the time of APSGN diagnosis, the blood urea nitrogen and creatinine levels were higher (P < 0.001) and the eGFR was lower (P< 0.001) in individuals with RPGN compared to those without RPGN. Furthermore, AKI was found in 41.2% of patients with RPGN at admission, with AKI stage 3 in 23.5%, compared to 4.5% in the other group. However, there was no significant correlation between hypertension (P=0.879), nephrotic range proteinuria (P=0.467), serum albumin levels (P=0.502), C-reactive protein (CRP) (P=0.099), reduced C3 level (P=0.453), and the course of RPGN. Kidney biopsy was performed on all patients with RPGN showing endocapillary proliferation in all of the patients; the number of glomerular crescents was > 50% in four (23%), and < 50% in eight (47%). In the group with RPGN, 17% needed kidney replacement therapy, 88.2% needed methylprednisolone pulse (P < 0.001), and 47.1% needed cyclophosphamide pulse (P = 0.008). The mean hospitalization time was higher in the group with RPGN (12 days) than in the group without RPGN (eight days) (P = 0.008). CKD progressed in only one (5%) of the 17 patients.

**Table 2:** Comparison of Clinical and Laboratory Characteristics Between Acute Post-streptococcal Glomerulonephritis (APSGN) Patients With and Without Rapidly Progressive Glomerulonephritis (RPGN) AKI: Acute kidney injury, eGFR: estimated Glomerular Filtration Rate

Variable	Without RPGN (N = 66)	With RPGN (N = 17)	Test Statistic	P-value
Age, years (mean ± SD)	8.35 ± 3.61	8.29 ± 3.55	t = 0.056	0.956
Sex, n (%):			χ² = 1.24	0.265
─ Male	48 (72.7%)	10 (58.8%)		
─ Female	18 (27.3%)	7 (41.2%)		
Hypertension, n (%)	44 (66.7%)	11 (64.7%)	χ² = 0.02	0.879
Blood urea nitrogen, mg/dL (median [IQR])	5.15 [3.4–9.38]	12.2 [7.3–16.7]	U = 217.5	< 0.001
Serum creatinine, mg/dL (median [IQR])	0.70 [0.50–1.13]	1.60 [0.84–2.38]	U = 208.0	< 0.001
AKI, n (%)	6 (9.1%)	7 (41.2%)	χ² = 9.31	0.004
AKI Stages, n (%)			Fisher’s exact	0.005
─ Stage 1	1 (1.5%)	2 (11.8%)		
─ Stage 2	2 (3.0%)	1 (5.9%)		
─ Stage 3	3 (4.5%)	4 (23.5%)		
eGFR, mL/min/1.73 m² (mean ± SD)	99.03 ± 52.74	41.77 ± 33.06	t = 4.33	< 0.001
Nephrotic syndrome, n (%)	21 (31.8%)	7 (41.2%)	χ² = 0.53	0.467
Serum albumin, g/dL (mean ± SD)	3.32 ± 0.79	3.16 ± 0.97	t = 0.68	0.502
CRP, mg/L (median [IQR])	6 [2–17.3]	10 [5–42]	U = 342.0	0.099
Reduced C3, n (%)	57 (86.4%)	13 (76.5%)	χ² = 0.56	0.453
Kidney biopsy performed, n (%)	25 (37.9%)	17 (100%)	χ² = 22.59	< 0.001
Kidney biopsy findings:			Fisher’s exact	0.088
─ Glomerular crescents	4 (16%)	6 (37.5%)		
─ Fibrosis	0 (0%)	1 (6.3%)		
Kidney replacement therapy, n (%)	2 (3%)	3 (17.6%)	χ² = 3.64	0.056
Methylprednisolone therapy, n (%)	21 (31.8%)	15 (88.2%)	χ² = 17.31	< 0.001
Cyclophosphamide therapy, n (%)	10 (15.2%)	8 (47.1%)	χ² = 7.08	0.008
Hospitalization time, days (mean ± SD)	8.17 ± 5.47	12.00 ± 3.76	t = -2.76	0.008

## Discussion

APSGN is an immune complex-mediated kidney disease caused by β-hemolytic Group A streptococci [11,12]. It is the third most common glomerulonephritis in the Moroccan series reported by Ramdani et al. [13]. Its incidence is difficult to assess due to the frequency of subclinical forms [14]. Still, globally, the incidence of APSGN has drastically decreased in recent decades, with an estimated incidence of 0.3/100,000/year in children [15]. Nevertheless, in developing countries, it was between 9.5 (low estimate) and 28.5 (high estimate) cases per 100,000 people due to poor hygiene, poverty, and crowded living conditions [16,17]. APSGN can arise in epidemic outbreaks, clusters, or isolated cases [17]. It is essential to note that APSGN epidemic cases are frequently reported in conjunction with cutaneous diseases [15]. In Australian cohorts, skin infections were the most prevalent cause of APSGN, which is in line with the general knowledge that impetigo is more common among Aboriginal and Torres Strait Islander children than pharyngitis [4]. This pattern of infection is like that seen in South Africa [1]. In contrast, other countries in North Africa, such as Tunisia, have found 73.6% of pharyngitis before APSGN [14], which is similar to our study (66.3%) (Figure 3). This disease affects children between two and 12 years of age and males more than females [16]. One possible explanation for the increased frequency of APSGN in the pediatric population might be the smaller pore size in children's glomerular basement membrane (GBM), which impedes the renal clearance of immune complexes. Conversely, the low incidence in the first two years of life might be due to a lower incidence of streptococcal infections, immaturity of the immune system, and a reduced capacity to form immunological complexes in this group [15]. The latent period between infection and APSGN is seven to 10 days for upper respiratory infections, two to four weeks for skin infections, and specifically 10 days in our cohort [17]. Asymptomatic disease (microscopic hematuria and reduced serum complement levels) is four to five times more prevalent than clinical disease [17]. In children, APSGN is the most common cause of acute transitory hypertension (30-90%); in 66% of our patients, it’s related to the retention of sodium and water in the extracellular space [18]. Maalej et al. [14] found a higher risk of developing high blood pressure linked to creatinine > 56.35 mmol/L in APSGN. In addition, Ge et al. [18] also demonstrated that hypertension (55.0% vs. 25.8%), AKI (50.0% vs. 17.2%) were significantly related to APSGN with nephrotic syndrome (APSGN-NS) patients compared to APSGN-no-NS patients. Yusof et al. [5] showed that the only factors substantially correlated with AKI were age and gross hematuria: it was more prevalent in those who were older and had gross hematuria, and the median time for AKI resolution was 18.0 days. It was also reported that AKI in APSGN was associated with the deposition of IgG and C3 in glomeruli, the severity of endocapillary proliferation, and interstitial damage. On the other hand, there was a statistical correlation between the more severe pathological grade and the lower C3 fraction [18]. In terms of complement level, CH50 and C3 are somewhat lower in 90% of cases during the first two weeks of the presentation [5]. A new complement activation method has been presented recently. In 31 out of 34 children with APSGN, autoantibodies against factor B, a part of the alternative pathway C3 convertase, were discovered by Chauvet et al. [19]. Anti-factor B antibody may help to determine the indication of kidney biopsy in case of nephritic syndrome with low C3 levels, helping to prevent the misdiagnosis of C3 glomerulopathy. This renal biopsy is rarely performed in children with typical APSGN, particularly in epidemic situations [17]; 24.4% of APSGN biopsies showed immune complex deposits together with crescentic glomerulonephritis, which was like the cohort of Abugrain et al. [1].

The frequency of occurrence of RPGN during post-infectious APSGN found in different series is low: nine cases out of 26 (7%) in the series by Baldwin et al. [19]; one case out of 35 (2.8%) in the Hinglais et al. series [20]; and seven cases out of 93 (7%) in the series by Abugrain et al. [1].

Karakaya et al. [21] found 12.4% of RPGN in their series and showed that patients with RPGN had substantially higher levels of CRP, platelet-to-lymphocyte ratio, CRP/albumin ratio, and erythrocyte sedimentation rate at onset (P < 0.05). and nephrotic range proteinuria was significantly correlated with the duration of RPGN (P = 0.024). Although, in our study, hypertension (P=0.879), nephrotic range proteinuria (P=0.467), serum albumin levels (P=0.502), CRP (P=0.099), and reduced C3 level (P=0.453) were not considered as factors predicting RPGN (Table 3).

**Table 3:** comparison of clinical and laboratory predictors of rapidly progressive glomerulonephritis (RPGN) * : number (percentage %), AKI: Acute kidney injury, eGFR: Estimated Glomerular Filtration Rate

Age, mean ± SD		D. Karakaya et al [20] (N = 19)	M. Jellouli et al. [19] (N = 27)	Our cohort (N = 17)
		8.50 ± 3.42	8,7	8.294 ± 3,549
Sex, n (%)	Male	-	17	10 (58.8)*
	Female	-	10	7 (41.2)*
Hypertension		-	15 (55)*	11 (64.7)*
Blood urea nitrogen, mg/dL (median [IQR])		-	18.7	12.2 [7.3-16.7]
Serum creatinine, mg/dL (median [IQR])		3.43 ± 1.57	3.4	1.6 [0.84-2.38]
AKI		-	27(100)*	7 (41.2)*
eGFR, mL/min/1.73 m2, mean ± SD		20.46 ± 12.17	-	41,765 ± 33.063
nephrotic proteinuria/syndrome		-	11 (40.7)*	7 (41.2)*
Serum albumin levels, g/dl		3.04 ± 0.48	-	3.16 ± 0.9701
CRP (mg/L)		67.02 ± 97.53	-	10 (5, 42)
Reduced C3 level		-	-	13 (76.5)*
Kidney biopsy		24 (15.7)*	26(96)*	17 (100)*
Kidney biopsy results	glomerular crescent	5(26.2)*	24(89)*	6 (37.5)*
	Fibrosis	-	3(11)*	1 (6.3)*
Kidney replacement therapy		9 (5.9)*	11(40)*	3 (17.6)*
Methylprednisolone pulse therapy		18 (11.8)*	18(66)*	15 (88.2)*
Cyclophosphamide pulse therapy		-	6(22)*	8 (47.1)*
Hospitalization time, days, mean ± SD		5.85 ± 5.54	-	12 ± 3.758

Since there is no specific therapy for APSGN, supportive care for symptoms and complications is the primary method of management. Fluid restriction, antihypertensives, diuretics, and, if required, dialysis or renal replacement therapy should be used to treat APSGN [22]. Loop diuretics have been used for more than 30 years to lower blood pressure and hasten the resolution of edema [17]. Eradication of streptococcal infection is a common strategy to prevent infection from spreading to household contacts [17], but according to a Cochrane analysis of 27 studies, antibiotic prophylaxis to avoid the risk of developing APSGN has no statistical significance [22].

In rare studies, methylprednisolone intravenous pulse therapy has alleviated crescentic APSGN with a rapidly progressive clinical course; nevertheless, the true efficacy of intravenous steroids and immunosuppression is still unproven [17,20,22,23]. In our study, all children with RPGN received methylprednisolone intravenous pulse. Nowadays, new therapeutic approaches, such as immunoadsorption, have demonstrated potential for therapy for PSGN. Furthermore, recent studies found that CD28-B7 inhibition decreased the generation of autoantibodies and cellular glomeruli infiltration, prevented target organs, and stopped PSGN from developing [5]. Future research is still needed to determine if immunosuppressive therapies are helpful.

In children, APSGN often has a favorable course, despite children with crescentic glomerulonephritis who can develop kidney failure [1,17,18,24]. The long-term progression of end-stage renal disease is rare in children with APSGN. Research conducted in Aboriginal Australian communities showed that individuals with a history of APSGN are more likely to experience a higher frequency of estimated glomerular filtration rates <60 ml/min and albuminuria, and if APSGN is linked to diabetes and obesity, it may also increase the risk of developing chronic renal failure [17,25].

According to KDIGO guidelines, all patients after AKI should be assessed at three months because of their heightened risk of developing CKD [2]. Only 2% of children with APSGN will progress to end-stage renal failure [22]. However, the nephrotic reduction caused by the initial inflammation may give way to a more severe form of the disease. Years after APSGN, kidney damage may continue or worsen as a result of nephron hypertrophy, hyperperfusion, or secondary inflammation following an infection [22]. Ge et al. [18] didn’t find a difference in prognosis between patients with APSGN-SN and APSGN with no SN. However, persistent hypertension may be present in 3-6% of children with resolved APSGN [22]. Moreover, Rodriguez-Iturbe et al. [17] reported that, after the acute phase, 20% of patients presented with abnormalities and/or a decrease in glomerular filtration; they prospectively followed 110 children with APSGN for 15-18 years and showed an incidence of 3.0% arterial hypertension, 5.4% microhematuria, 0.9% azotemia, and 7.2% proteinuria. In Iran, 94 children with acute glomerulonephritis were followed for seven years; 3.1% had proteinuria, 6.3% had microscopic hematuria, and none of the patients developed renal impairments or hypertension [26]. On the other hand, in Brazil, the incidence of hypertension was higher in APSGN groups than in control groups after 10 years of follow-up in children with APSGN, but there was no discernible difference in the assessment of renal function, which includes serum creatinine, cystatin C, eGFR, albuminuria, and hematuria [27]. In Hoy et al.'s study [25], children with a history of APSGN five years prior had more proteinuria and poorer glomerular filtration rates. Also Utari et al. [28] determined that in APSGN, severe proteinuria along with hypoalbuminemia and macroscopic hematuria are the predictive indicators that most strongly indicate chronic glomerulonephritis. Other studies found that a higher level of CRP, hypoalbuminemia, and hypocomplementemia are linked to a more severe clinical presentation and a more severe APSGN [22].

Vivante et al. [22] showed the existence of nephrotic syndrome, renal insufficiency, and crescent on biopsy results as indicators of a poor long-term prognosis for APSGN. Furthermore, certain histological signs may indicate a severe prognosis. Wong et al. [23] showed that progression to chronic end-stage renal failure was more prevalent in the APSGN with cellular crescents. Jellouli et al. [20] reported that five of 27 children with rapidly progressive post-infectious glomerulonephritis progressed to chronic renal failure despite aggressive treatment. The presence of anuria and massive proteinuria are factors with a poor renal prognosis [20]. There is a significant correlation between the percentage of glomeruli affected and progression to renal failure. The heterogeneity and poor prognosis of GNRP in children have led to numerous therapeutic trials, but there are no specific guidelines for them.

In our cohort, the median length of hospital stay was eight days; however, Limm-Chan et al. [3] reported that the average length of hospitalization was 4.7 days, although it may be as long as 21 days. Higher creatinine and lower bicarbonate levels were linked to longer hospitalization.

It is important to recognize the many limitations of our study that affect the strength and generalizability of our conclusions. The retrospective nature of the study and the single-center design limit the application of the results to patients with different demographics and healthcare practices. Furthermore, because of the lack of biopsy confirmation, there is uncertainty about the diagnosis. Such uncertainty could lead to misclassification and affect the accuracy of the reported association between clinical features and outcomes.

The absence of multivariate analysis further limits the possibility to control for confounding variables and draw causal conclusions about risk factors. Finally, the one-year follow-up period is important, but it cannot provide an accurate assessment of long-term kidney disorders like chronic kidney disease or recurrence.

Thus, after the acute period of their illness, it is necessary to monitor these children to make sure that their renal function, proteinuria and complement levels return to normal for a long time.

## Conclusions

Even while APSGN is on the decline, it is still a health concern in emerging and less developed nations. In children, the outcome is favorable, except for those who developed crescentic glomerulonephritis, which often leads to renal failure. Considering the severity of APSGN and AKI in children, the absence of a strong follow-up strategy is very alarming. Proactive surveillance is essential following an APSGN, as the early identification of chronic renal impairment could halt the long-term progression of CKD.
